# The dynamics analysis of Gompertz virus disease model under impulsive control

**DOI:** 10.1038/s41598-023-37205-x

**Published:** 2023-06-22

**Authors:** Linjun Wang, Aiqing She, Youxiang Xie

**Affiliations:** 1 Hubei key Laboratory of Hydroelectric Machinery Design and Maintenance, College of Mechanical and Power Engineering, China Three Gorges University, Yichang, 443002 Hubei People’s Republic of China; 2 School of Mathematics, Hunan University, Changsha, 410082 Hunan People’s Republic of China; 3 College of Science Technology, China Three Gorges University, Yichang, 443002 Hubei People’s Republic of China

**Keywords:** Computational biology and bioinformatics, Ecology

## Abstract

The pandemic of Gompertz virus disease remains a pressing issue in agricultural production. Moreover, the dynamics of various infectious diseases is usually investigated by the method of mathematical modelling. A new mathematical model for dynamics on Gompertz virus disease impulsive system is proposed and analyzed in this paper. We prove the dynamic characteristics about the permanence and globally exponential stability of Gompertz virus disease model. Moreover, we also give the sufficient condition that one positive solution which satisfies $E(t) \ge q_2$ if $R_{1*}> 1$ exists. Eventually, numerical simulations are utilized to validate the validity of the theoretically analyzed conclusion in this paper.

## Introduction

It is well-known that a nonlinear system model usually has a rich dynamic behavior which has been payed attention to by many researchers^1–6^. Moreover, in recent years, many researchers have paid attention to biological control^7–16^.

Due to many cases that we need to describe the biological dynamics with sudden changes as well as other phenomena, impulsive differential equations play important part^17–19^. In consideration of hybrid nature, different kinds of evolutionary processes in real-world life are usually described by impulsive systems, and these evolutionary processes usually state a big sudden change at some moments^20–23^. Some researchers stated that differential equations with impulse do well in describing biological control, and thought that differential equations with impulsive effect are more practical than ordinary differential equations in stating practical problems^24,25^. Liang et al.^26^ proposed the impulsive control of Leslie predator-prey model, and investigated the dynamic properties of the proposed mathematical biology model. The herbivore-plankton model with cannibalism was proposed by Fang et al.^27^, and the corresponding heteroclinic bifurcations and three order-1 periodic orbits were analyzed. A new impulsive state feedback model was proposed, and the management strategy with impulsive control was developed^28^. Wang and Chen^29^ proposed a new microbial pesticide model and analyzed the condition of the existence of corresponding periodic orbit. Li and Chen^30^ investigated the dynamics of the impulsive turbidostat model, and also studied the characteristic of the proposed mathematical biology model. Li et al.^31^ proposed a new water hyacinth fish impulsive control model, and investigated the local stability and global dynamics of the proposed ecological system. Li et al.^32^ proposed a new Filippov predator-prey model, and analyzed the corresponding dynamics using the Filippov theory knowledge. Qin et al.^33^ proposed a new non-smooth Filippov ecosystem with group defense and also studied the stability of equilibria and the bifurcation phenomenon numerically. Discontinuous plant disease models with a non-smooth separation line were investigated and discussed by Li et al.^34^. Khan et al., proposed some new hepatitis B epidemic models and novel corona virus disease models and investigated their dynamic characteristics^35–40^.

In fact, exploiting viruses and the release of pest population simultaneously, some research studies have been attached to devote the control of pests^9,41^. First of all, introducing pathogens into the pest population is expected to produce an epidemic and then become endemic. Moreover, the infected pests are released to the periodic impulsive pest population. However, the crops will not be affected by the infected pest. The vulnerable pests are infected by direct contact with infected pests or being exposed to an infected environment. Then the pest population and its death will be affected to some extent^42,43^. In recent years, the pandemic of Gompertz virus disease remains a pressing issue in epidemiological ecosystem. Mathematical modelling is an important method in investigating the dynamic characteristics of epidemiological ecosystem. Additionally, due to the hybrid nature, in order to well describe different kinds of real-world evolutionary processes, impulsive systems play important role because it states sudden change at some moments. This article will try to develop a new epidemiological ecosystem with impulsive effect and investigate its dynamics for bio-control. Additionally, it is assumed that the pest population will grow according to the logic curve without the disease^9,44,45^. Then we investigate the global attractiveness and permanence of the proposed model.

The remainder of this paper is provided as follows. In section “Mathematical model”, the Gompertz virus disease model with impulsive effect is established. In section “Global attractiveness”, utilizing the comparison theorem and Floquet theory of impulsive differential equation, the global attractiveness of the proposed Gompertz virus disease model is strictly proved. In section “Uniform persistence”, we obtain the criteria for the uniform persistence of the proposed Gompertz virus disease model. In section “Numerical simulations”, many numerical results are given to prove the validity of the obtained results. Finally, some conclusions are summarized.

## Mathematical model

In this section, we will present the newly developed Gompertz virus disease model with impulsive effect.

The total population of insect pest is denoted by *N*(*t*),  and it is divided into five subgroups such as *I*(*t*),  *E*(*t*), *S*(*t*), *R*(*t*) and *A*(*t*). The susceptible pest is denoted by *S*(*t*);  *E*(*t*) denotes the exposed pest; the infected (symptomatic) pest is denoted by *I*(*t*);  *A*(*t*) represents the asymptotically infected pest; the recovered or the removed pest is denoted by *R*(*t*). The class *M*(*t*) denotes the reservoir or the seafood place or market.

In this paper, the impulsive introduction of disinfectants and the pulse vaccination strategy in the environment are investigated for the control of the Gompertz virus epidemic, and we also assume that the proposed model satisfies:

1. $\mu$ and $\Pi$ respectively denotes the natural death rate and the birth rate of the pest.
2. The susceptible pest *S*(*t*) acquires Gompertz virus infection by ingesting environmental virus from the contaminated reservoir or seafood place or market, through the term given by $\beta \frac{{S(t)}}{{K + S(t)}},$ where $\beta$ is the disease transmission coefficient.
3. The proportion of asymptomatic infection is denoted by $\theta$. The transmission rate of the infected is denoted by $\rho .$ $\varpi$ denotes the transmission rate after completing the incubation period. *K* represents the pathogen concentration.
4. $\tau _1$ and $\tau _2$ respectively denote the pest in *I*(*t*) and *A*(*t*) joining *R*(*t*) with the removal or recovery rate, and Gompertz virus induced mortality rate of infected individuals is denoted by $d_1$.
5. $\eta M(t)E(t)$ denotes the susceptible pest infected after the interaction with *M*(*t*), in which the disease transmission coefficient between *S*(*t*) and *E*(*t*) is represented by $\eta .$
6. The parameters $\lambda _1$ and $\lambda _2$ respectively denote the infected symptomatic and the asymptomatically infected contributing the Gompertz virus into the seafood market *M*(*t*). The removing rate of the Gompertz virus from the seafood market *M*(*t*) is given by the rate *d*. The natural growth rate of the Gompertz virus is denoted by *b*. We assume $b < d$ through this paper.
7. The parameter $\theta _1(0< \theta _1<1)$ denotes the part of susceptible pest inoculated by the vaccine at times $t=nT,$ in which *n* is a positive integer, and the impulsive period between two consecutive pulse vaccinations is denoted by *T*. *nT* represents the pulse vaccination at the time of multiple periods. *R*(*t*) represents the temporary immunity which contains the recovered individual and the vaccinated individual.
8. The death of the Gompertz virus dues to the environmental sanitation. Therefore, considering the pulse inoculation of disinfectant in the environment at the time of *nT*,  the death rate of the virus is denoted by $\xi (0<\xi <1).$ The model is expressed as:
  1 $$\begin{aligned} \left\{ {\begin{array}{lll} {\left. {\begin{array}{lll} {\begin{array}{lll} {\dot{S}(t) = \Pi - S(t)M(t) E(t)\eta -\frac{{S(t)\beta E(t)}}{{K+S(t)}} - \mu S(t) } \\ {\dot{E}(t) = \frac{{S(t)\beta E(t)}}{{K+S(t)}}-E(t)\mu - E(t)\theta \rho - E(t)(1-\theta )\varpi +\eta E(t)M(t)S(t)} \\ {\dot{I}(t) = (1 - \theta )\varpi E(t) - (\tau _1 + \mu + d_1 )I(t)} \\ \end{array}} \\ {\begin{array}{lll} {\dot{A}(t) = \theta \rho E(t) - (\tau _2 + \mu )A(t)} \\ {\dot{R}(t) = \tau _1 I(t) + \tau _2 A(t) - \mu R(t)} \\ {\dot{M}(t) = M(t)(b - d)+\lambda _1 I(t) + \lambda _2 A(t)} \\ \end{array}} \\ \end{array}} \right\} \;\;\;n \in N^ +, t \ne nT.} \\ {} \\ {\left. {\begin{array}{lll} {\begin{array}{lll} {S(t^ + ) = S(t)(1 - \theta _1 )} \\ {E(t^ + ) = E(t)} \\ {I(t^ + ) = I(t)} \\ \end{array}} \\ {\begin{array}{lll} {A(t^ + ) = A(t)} \\ {R(t^ + ) = \theta _1S(t)+R(t)} \\ {M(t^ + ) = M(t)(1 - \xi )} \\ \end{array}} \\ \end{array}} \right\} \;\;\; n \in N^ +, t=nT.} \\ \end{array}} \right. \end{aligned}$$

For (1), all the coefficients are greater than 0. Based on

2 $$\begin{aligned} \dot{N}(t) = \Pi - \mu N(t) - d_1 I(t), \end{aligned}$$

then the population size will change with *t*. If $A(t) =I(t) =E(t) = 0$, we have $\mathop {\lim }\limits _{t \rightarrow \infty } N(t) = \frac{\Pi }{\mu }.$

Similarly, we have

$$\begin{aligned} \dot{M}(t) = \lambda _2 A(t) - (d-b)M(t)+\lambda _1 I(t) \Rightarrow \mathop {\lim }\limits _{t \rightarrow \infty } \sup M(t) \le \frac{{\Pi (\lambda _1 + \lambda _2 )}}{{\mu (d-b)}}. \end{aligned}$$

Then we have that when *t* takes the sufficiently large value,

$$\begin{aligned} M(t) \le \frac{{\Pi (\lambda _1 + \lambda _2 )}}{{\mu (d-b)}}, N(t) \le \frac{\Pi }{\mu }. \end{aligned}$$

By replacing $N(t)-S(t)-E(t)-I(t)-A(t)$ with *R*(*t*),  then

3 $$\begin{aligned} \left\{ {\begin{array}{lll} {\left. {\begin{array}{lll} {\begin{array}{lll} {\dot{S}(t) = \Pi -\mu S(t)-\frac{{S(t)\beta E(t)}}{{K+S(t)}}}- E(t)S(t)M(t)\eta \\ {\dot{E}(t) = \frac{{\beta E(t)S(t)}}{{S(t) + K}}-E(t)\mu - E(t)\theta \rho - (1 - \theta )\varpi E(t) + \eta E(t)M(t)S(t)} \\ {\dot{I}(t) =- (\tau _1 + \mu + d_1 )I(t)+ (1 - \theta )\varpi E(t) } \\ \end{array}} \\ {\begin{array}{lll} {\dot{A}(t) = - (\tau _2 + \mu )A(t)+\theta \rho E(t)} \\ {\dot{N}(t) = - \mu N(t) - d_1 I(t)+\Pi }\\ {\dot{M}(t) =M(t)(b - d)+ I(t)\lambda _1 + A(t)\lambda _2} \\ \end{array}} \\ \end{array}} \right\} \;\;\;n \in N^+,t\ne nT} \\ {} \\ {\left. {\begin{array}{lll} {\begin{array}{lll} {(1-\theta _1)S(t)=S(t^+)}\\ {E(t)=E(t^+)}\\ {I(t)=I(t^+)}\\ \end{array}} \\ {\begin{array}{lll} {A(t)=A(t^+)} \\ {N(t)=N(t^+)} \\ {M(t)(1 - \xi )=M(t^+)}\\ \end{array}} \\ \end{array}} \right\} \;\;\;t = nT,n\in N^+.} \\ \end{array}} \right. \end{aligned}$$

We provide the initial condition for (3) as:

* $$\begin{aligned} \phi (\omega )=(\phi _1(\omega ),\phi _2(\omega ),\phi _3(\omega ),\phi _4(\omega ),\phi _5(\omega ),\phi _6(\omega ))\in R_+^6,\phi _i(0)>0,i=1,2,\ldots ,6. \end{aligned}$$

Next, we will study the dynamic properties of (3). For biological considerations, (3) is investigated in the following closed set and this set is given as

$$\begin{aligned} \begin{array}{l} \Omega = \{ \left( {S(t),E(t),I(t),A(t),N(t),M(t)} \right) \in R_ + ^6:\\ S(t) \ge 0,E(t) \ge 0,I(t) \ge 0,A(t) \ge 0,N(t) \ge 0,M(t) \ge 0, \\ 0 \le S(t) + E(t) + I(t) + A(t) \le \frac{\Pi }{\mu },N(t) \le \frac{\Pi }{\mu },M(t) \le \frac{{\Pi (\lambda _1 + \lambda _2 )}}{{\mu (d - b)}},d > b\} \begin{array}{*{20}c} {} &{} {} \\ \end{array}\\ \end{array} \end{aligned}$$

In fact, it is not difficult to check that $\Omega$ has a positively invariant property about (3). For further discussion, some important assumptions and definitions are given as follows.

We assume that the fixed solution and the arbitrary solution of (3) are respectively given by

$$\begin{aligned} x^* (t) =(S^*(t),E^*(t),I^*(t),A^*(t),N^*(t),M^*(t)), \end{aligned}$$

and

$$\begin{aligned} x(t) =(S(t),E(t),I(t),A(t),N(t),M(t)). \end{aligned}$$

### Definition 1

$\forall$ $x_0$, $\exists$ $M \ge m > 0,$ we have that when *t* takes the sufficiently large value, $x(t)\in [m, M].$ Then (3) has the uniform persistence.

### Definition 2

For arbitrary $x_0$, If $\mathop {\lim }\limits _{t \rightarrow \infty } \left| {x(t)-x^*(t)}\right| = 0$, then $x^*(t)$ has the global attractivity.

### Definition 3

$\forall$ $\eta >0,$ and $\varepsilon >0,$ $\left| {x^*(t)-x(t)} \right| < \varepsilon$ when there is $\delta = \delta (t_0 ,\varepsilon ,\eta ) > 0.$ Moreover, if $\left| {t_0-t} \right| > \eta$, and $t\in [ t_0,+\infty ],$ then $\left| {x_0 - x^*_0 } \right| < \delta .$ Then $x^*(t)$ has the characteristic of stability.

### Definition 4

When the fixed solution $x^*(t)$ has the characteristic of stability and global attractivity, then $x^*(t)$ is called globally asymptotical stable. If there is the disease-free periodic solution for (3) which also has the characteristic of globally asymptotical stability, the Gompertz virus will finally disappear.

### Definition 5

(Equicontinuty) For $\forall \varepsilon >0,$ if we can find a positive parameter $\delta ,$ which satisfies $|x(t_2)-x(t_1 )|<\varepsilon$ holds true when $t_i(i=1,2)\in [0,l]$ and $|t_1-t_2 |<\delta ,$ *x*(*t*) is said to be equicontinuous when $0\le t\le 1.$

For further analysis conveniently, the following lemma is given as follows.

### Lemma 1

^46^ We consider

4 $$\begin{aligned} \left\{ {\begin{array}{llll} {\dot{u}(t) = - \mu u(t)+\Pi ,} {t \ne nT} \\ {u(t^+) = u(t)(1 - \theta _1 ),}{n \in N^ +,t = nT} \\ \end{array}} \right. \end{aligned}$$

where $$0<\theta _1<1,$$ $$\mu >0$$ and $$\Pi >0.$$

Thus we can obtain that for $t\in [nT,(n + 1)T],$ the periodic solution is given as

$$\begin{aligned} \begin{array}{l} u^* (t) = \frac{\Pi }{\mu } + (u^* - \frac{\Pi }{\mu })e^{ - \mu (t - nT)} \\ \;\;\;\;\;\;\;\; \;= \frac{\Pi }{\mu }(1 - \frac{{\theta _1 e^{ - \mu (t - nT)} }}{{1 - (1 - \theta _1 )e^{ - \mu T} }})\;\; \\ \end{array} \end{aligned}$$

where

$$\begin{aligned} u^* = \frac{\Pi }{\mu }\frac{{(1 - e^{ - \mu T} )(1 - \theta _1 )}}{{1 - (1 - \theta _1 )e^{ - \mu T} }} \end{aligned}$$

exists and is globally asymptotically stable, unique and positive.

## Global attractiveness

In this section, we will discuss the global attractiveness of the Gompertz virus disease model with impulsive effect (3).

$$\forall$$ $$t\in [0,+\infty ],$$

$$\begin{aligned} M(t)=I(t)=A(t)=E(t)=0. \end{aligned}$$

It means that the asymptotically infectious individuals permanently die out. Based on this prerequisite, we can transform (3) as:

5 $$\begin{aligned} \left\{ \begin{array}{lll} \left. \begin{array}{rcl} \dot{S}(t)&{}=&{}\Pi - \mu S(t),\\ \dot{N}(t)&{}=&{}\Pi - \mu N(t),\ \ \end{array}\right\} \ \ \ n \in N^ +, t\ne nT\\ \\ \left. \begin{array}{lll} S(t^ + ) &{}=&{}(1 - \theta _1 )S(t)\\ N(t^ + ) &{}=&{}N(t), \end{array}\right\} \ \ \ n \in N^+, t = nT\\ \end{array}\right. \end{aligned}$$

Then we can obtain

$$\begin{aligned} \mathop {\lim }\limits _{t \rightarrow \infty } N(t) = \frac{\Pi }{\mu }. \end{aligned}$$

Next, we will prove that for the susceptible *S*(*t*),  there is one periodic solution. Firstly, we consider

6 $$\begin{aligned} \left\{ {\begin{array}{*{20}c} {\dot{S}(t) = \Pi - \mu S(t), n \in N^+, t \ne nT} \\ {S(t^ + ) = (1 - \theta _1 )S(t), n \in N^+, t = nT.} \\ \end{array}} \right. \end{aligned}$$

It follows from Lemma 1 that a unique globally asymptotical periodic solution of (6) exists, and it is given as

7 $$\begin{aligned} S^*(t) = \frac{\Pi }{\mu }\left( {1 - \frac{{\theta _1 e^{ - \mu (t - nT)} }}{{1 - (1 - \theta _1 )e^{ - \mu T} }}} \right) , t\in (nT,(n + 1)T].\end{aligned}$$

Therefore, there exists one infection-free periodic solution which is given by $(S^*(t),0,0,0,\frac{\Pi }{\mu },0).$

### Theorem 1

For system (3), $\left( S^*(t),0,0,0,\frac{\Pi }{\mu },0 \right)$ has global attractivity for $R_1^* < 1$, in which

$$\begin{aligned} R_1^*= & {} \frac{{\left( {\frac{\beta }{{K + \zeta }} + \frac{{\eta \Pi }}{{\mu (d-b)}}(\lambda _1 + \lambda _2 )} \right) \zeta }}{{(1 - \theta )\varpi + \theta \rho + \mu }}, \\ \zeta= & {} \frac{{\Pi \left( {e^{\mu T} - 1} \right) }}{{\mu (e^{\mu T} + \theta _1 - 1)}}. \end{aligned}$$

### Proof

Since $R_1^*< 1$, $\varepsilon _1^{} > 0$ can be chosen such that

8 $$\begin{aligned} \left( {\frac{\beta }{{K + \zeta + \varepsilon _1 }} + \frac{{\eta \Pi (\lambda _1 + \lambda _2 )}}{{\mu (d-b)}}} \right) \left( {\zeta + \varepsilon _1 } \right) < (1 - \theta )\varpi + \theta \rho + \mu . \end{aligned}$$

According to $\dot{N}(t) \le - \mu N(t)+\Pi ,$ then there exists $n_1>0$ satisfying the following inequality

9 $$\begin{aligned} N(t) \le \frac{\Pi }{\mu },t > n_1 T. \end{aligned}$$

By (3), then

$$\begin{aligned} \dot{S}(t) < - \mu S(t)+\Pi . \end{aligned}$$

When $t > n_1 T$ and $n > n_1,$ we investigate

10 $$\begin{aligned} \left\{ {\begin{array}{lll} {\dot{u}(t) = - \mu u(t)+\Pi ,} {t \ne nT} \\ {u(t^ + ) = u(t)(1 - \theta _1 ),}{n \in N^ +, t = nT.} \\ \end{array}} \right. \end{aligned}$$

For system (10), we can obtain the globally asymptotical stable periodic solution as $u^*(t) = S^*(t),$ utilizing Lemma 1, and also there is $n_2> n_1$ such that when $n > n_2$,

11 $$\begin{aligned} S(t)< S^*(t) + \varepsilon _1 \le \frac{{\Pi (e^{\mu T} - 1)}}{{\mu (e^{\mu T} + \theta _1 - 1)}} + \varepsilon _1 = \delta ,nT < t \le (n + 1)T. \end{aligned}$$

Then

$$\begin{aligned} \dot{E}(t) \le \left[ {\frac{{\beta \delta }}{{\delta + K}} + \eta \delta \frac{{\Pi \left( {\lambda _1 + \lambda _2 } \right) }}{{\mu \left( {d - b} \right) }} - (1 - \theta )\varpi - \theta \rho - \mu } \right] E(t),t> nT,n > n_2. \end{aligned}$$

Thus

12 $$\begin{aligned} \mathop {\lim }\limits _{t \rightarrow \infty } E(t) = 0. \end{aligned}$$

Therefore, for $\forall \varepsilon _2> 0$, there is $n_3>n_2$, satisfying $E(t) < \varepsilon _2$ when $t > n_3 T$.

Then, according to (3), we can obtain that if $t> nT,n> n_3,$

$$\begin{aligned} \dot{I}(t) \le - (\tau _1 + \mu + d_1 )I(t)+ (1 - \theta )\varpi \varepsilon _2 \end{aligned}$$

i.e.

$$\begin{aligned} I(t) < \left[ {I(nT) - \frac{{\varpi \varepsilon _2 \left( {1 - \theta } \right) }}{{\tau _1 + \mu + d_1 }}} \right] e^{ - (t - nT)(d_1 + \tau _1 + \mu )} + \frac{{\left( {1 - \theta } \right) \varpi \varepsilon _2 }}{{\tau _1 + \mu + d_1 }}. \end{aligned}$$

Hence,

13 $$\begin{aligned} \mathop {\lim }\limits _{t \rightarrow \infty } I(t) = 0. \end{aligned}$$

Similarly, for $\forall \varepsilon _3 > 0$, there is $n_4 > n_3$, satisfying

$$\begin{aligned} I(t) < \varepsilon _3 \end{aligned}$$

when $$t > n_4 T$$.

Exploiting the fourth one of (3), the following inequality can be obtained as

$$\begin{aligned} \dot{A}(t) \le \theta \rho \varepsilon _3 - (\tau _2 + \mu _1 )A(t) \end{aligned}$$

for all $$t> nT (n > n_4).$$

Obviously

14 $$\begin{aligned} \mathop {\lim }\limits _{t \rightarrow \infty } A(t) = 0. \end{aligned}$$

Therefore, for arbitrary $\forall \varepsilon _4 > 0$, there is $n_5>n_4$ satisfying $A(t) < \varepsilon _4$ when $t > n_5 T.$

Next, according to the sixth one of (3), we get

$$\begin{aligned} \left\{ {\begin{array}{*{20}c} {\dot{M}(t) \le \lambda _1 \varepsilon _3 + \lambda _2 \varepsilon _4 - dM(t),t \ne nT} \\ {M(t^ + ) = M(t)(1 - \xi ), n > n_5, t = nT.} \\ \end{array}} \right. \end{aligned}$$

Obviously,

15 $$\begin{aligned} \mathop {\lim }\limits _{t \rightarrow \infty } M(t) = 0. \end{aligned}$$

Therefore, for $\forall \varepsilon _5>0$, there is $n_6 > n_5$ satisfying $M(t) < \varepsilon _5$ at the case $t> n_6 T.$

Eventually, we can obtain

$$\begin{aligned} \dot{N}(t) \ge \Pi - \mu N(t) - d_1 \varepsilon _3. \end{aligned}$$

when $$t > n_6 T.$$

When $t > n_6 T$, the following comparative model will be investigated.

16 $$\begin{aligned} \dot{Z}(t) = \Pi - \mu Z(t) - d_1 \varepsilon _3, \end{aligned}$$

Obviously,

$$\begin{aligned} \mathop {\lim }\limits _{t \rightarrow \infty } Z(t) = \frac{{\Pi - d_1 \varepsilon _3 }}{\mu }. \end{aligned}$$

Exploiting the corresponding lemma of^25^, we can obtain that if $t> n_7 T$, there is $n_7 > n_6$ such that

17 $$\begin{aligned} N(t) \ge \frac{{\Pi - d_1 \varepsilon _3 }}{\mu } - \varepsilon _6. \end{aligned}$$

Because $\varepsilon _3>0$ and $\varepsilon _6>0,$ and they are extremely small, we can obtain by (9) and (17) that

18 $$\begin{aligned} \mathop {\lim }\limits _{t \rightarrow \infty } N(t) = \frac{\Pi }{\mu }. \end{aligned}$$

From (9) and (12)–(15), we can get the following conclusion:

For $\forall \varepsilon _7>0$, there is $n_8 > n_7$ satisfying

$$\begin{aligned} M(t)< \varepsilon _7, A(t)<\varepsilon _7, I(t)< \varepsilon _7, E(t) < \varepsilon _7, N(t) >\frac{\Pi }{\mu }, \end{aligned}$$

when $$t > n_8 T.$$

When $t > n_8 T$, it follows from (3) that

$$\begin{aligned} \dot{S}(t)\ge \Pi - \frac{{\beta \delta }}{{\delta + K}}\varepsilon _7 - \left( \mu + \eta \varepsilon _7^2 \right) S(t), \end{aligned}$$

when $$t > nT$$ and $$n > n_8.$$

Considering

19 $$\begin{aligned} \left\{ {\begin{array}{lll} {\dot{u}(t) \ge \Pi - \frac{{\beta \delta }}{{\delta + K}}\varepsilon _7 - \left( \mu + \eta \varepsilon _7^2 \right) S(t),t \ne nT} \\ {u \left( t^ + \right) = u(t)(1 - \theta _1 ), n\in N^+, t = nT, n>n_8,} \\ \end{array}} \right. \end{aligned}$$

based on Lemma 1, its unique asymptotical stable periodic solution can be computed as

$$\begin{aligned} \mathop u\limits ^ - (t) = \frac{{\Pi - \frac{{\beta \delta }}{{\delta + K}}\varepsilon _7 }}{{\mu + \eta \varepsilon _7^2 }} + \left( u^* - \frac{{\Pi - \frac{{\beta \delta }}{{\delta + K}}\varepsilon _7 }}{{\mu + \eta \varepsilon _7^2 }}_{} \right) e^{ - (t - nT) \left( \eta \varepsilon _7^2+\mu \right) } \end{aligned}$$

when $nT<t\le (n + 1)T,$ in which

$$\begin{aligned} u^* = \frac{{\Pi - \frac{{\beta \delta }}{{\delta + K}}\varepsilon _7 }}{{\mu + \eta \varepsilon _7^2 }}\frac{{\left( 1 - e^{ - \left( \mu + \eta \varepsilon _7^2 \right) T} \right) (1 - \theta _1 )}}{{1 - e^{ - \left( \mu + \eta \varepsilon _7^2 \right) T} (1 - \theta _1 )}}. \end{aligned}$$

According to^25^, there exists $n_9 > n_8$ satisfying

20 $$\begin{aligned} S(t) > \mathop u\limits ^ - (t) - \varepsilon _7 \end{aligned}$$

for

$$\begin{aligned} n > n_9, nT < t \le (n+ 1)T. \end{aligned}$$

Because $\varepsilon _7$ and $\varepsilon _1$ take the very small value, then we have

21 $$\begin{aligned} \mathop {\lim }\limits _{t \leftarrow \infty } S(t) = S^*(t) = \left( {1 - \frac{{\theta _1 \left( 1 - e^{ - \mu (t - nT)} \right) }}{{1 - \left( 1 - \theta _1 \right) e^{ - \mu T} }}} \right) \frac{\Pi }{\mu } \end{aligned}$$

when $$nT<t\le (n + 1)T.$$

By (12)–(15), (18) and (21), the infection-free periodic solution $(S^*(t),0,0,0,\frac{\Pi }{\mu },0)$ is the globally attractive. Based on Theorem 1, we can obtain some assertions as follows.

### Proposition 1

- The infection-free periodic solution which is given by $(S^*(t),0,0,0,\frac{\Pi }{\mu },0)$ is globally attractive, when
  $$\begin{aligned} \Pi \left( {\frac{{\beta \mu }}{{\Pi + \mu K}} + \frac{{\eta \Pi (\lambda _1 + \lambda _2 )}}{{\mu (d-b)}}} \right) < \mu ((1 - \theta ) + \theta \rho + \mu ); \end{aligned}$$
- If $$T<T^*$$ and
  $$\begin{aligned} \Pi \left( {\frac{{\beta \mu }}{{\Pi + \mu K}} + \frac{{\eta \Pi (\lambda _1 + \lambda _2 )}}{{\mu (d-b)}}} \right) > \mu ((1 - \theta ) + \theta \rho + \mu ), \end{aligned}$$
  $\left( S^*(t),0,0,0,\frac{\Pi }{\mu },0\right)$ is globally attractive, where
  $$\begin{aligned} \begin{array}{*{20}c} \begin{array}{l} T^* = \frac{1}{\mu }\ln \left( {1 + \frac{{\upsilon \theta _1 \mu }}{{\Pi - \upsilon \mu }}} \right) , \\ A = \eta \pi (\lambda _1 + \lambda _1 ), \\ B = \mu (d-b), \\ C = (1 - \theta )\varpi + \theta \rho + \mu , \\ \end{array} \\ {\upsilon = \frac{{ - \left( {B + KA - BC} \right) + \sqrt{\left( {B + KA - BC} \right) ^2 + 4KABC} }}{{2A}}}. \\ \end{array} \end{aligned}$$

### Proposition 2

If

$$\begin{aligned} \theta _1> \theta _1^*= \frac{{\left( \Pi - \mu \upsilon \right) \left( e^{\mu T} - 1\right) }}{{\mu \upsilon }}, \end{aligned}$$

$\left( S^*(t),0,0,0,\frac{\Pi }{\mu },0 \right)$ is globally attractive.

## Uniform persistence

In this section, we will discuss the sufficient conditions for the Gompertz virus disease model with impulsive effect. In order to obtain uniform persistence, the following lemma needs to be proved first.

### Lemma 2

If $R_{1*}> 1,$ then for arbitrary $t_0 > 0, E(t) < \mathop E\limits ^\_$ doesn’t hold true for all $t > t_0 ,$ where

$$\begin{aligned} R_{1*}^{}= & {} \frac{{\frac{{\beta \zeta }}{{K + \zeta }}}}{{(1 - \theta )\varpi + \theta \rho + \mu }}, \\ \zeta= & {} \frac{{\Pi \left( {e^{\mu T} - 1} \right) (1 - \theta _1 )}}{{\mu (e^{\mu T} + \theta _1 - 1)}}, \\ \mathop E\limits ^\_= & {} \frac{{\Pi \left( {e^{\mu T} - 1}\right) (1-\theta _1) }}{{(e^{\mu T} + \theta _1 - 1)\left( {(1 - \theta )\varpi + \theta \rho + \mu } \right) \left( {K + \zeta } \right) }} - \frac{\mu }{\beta }. \end{aligned}$$

### Proof

Herein we use the method of contradiction. We assume that there is $t_0 > 0,$ which satisfies when $t > t_0,$

22 $$\begin{aligned} E(t) < \mathop E\limits ^\_. \end{aligned}$$

When $$t_0<t,$$

23 $$\begin{aligned} \dot{I}(t)\le \mathop E\limits ^\_ (1 - \theta )\varpi -I(t)(\tau _1 + \mu + d_1 ) \end{aligned}$$

and

24 $$\begin{aligned} \dot{A}(t) \le \theta \rho \mathop E\limits ^\_ - (\tau _2 + \mu _1 )A(t). \end{aligned}$$

By (23) and (24), then $\exists \varepsilon ^* > 0$ and when $t_1 > t_0 ,$ then

$$\begin{aligned} I(t){} & {} \le \frac{{(1 - \theta )\varpi \mathop E\limits ^\_ }}{{\tau _1 + \mu + d_1 }} + \varepsilon ^ *, \\{} & {} \quad \frac{{\frac{{\beta (\zeta - \varepsilon ^* )}}{{k + \zeta - \varepsilon ^* }}}}{{(1 - \theta )\varpi + \theta \rho + \mu }} > 1, \end{aligned}$$

and

$$\begin{aligned} A(t) \le \frac{{\theta \rho \mathop E\limits ^\_ }}{{\tau _2 + \mu }} + \varepsilon ^ * \end{aligned}$$

for arbitrary $$t > t_1.$$

Thus, when $$t > t_1,$$

$$\begin{aligned} \dot{M}(t) \le \lambda _1 \frac{{(1 - \theta )\varpi \mathop E\limits ^\_ }}{{\tau _1 + \mu + d_1 }} + \lambda _2 \frac{{\theta \rho \mathop E\limits ^\_ }}{{\tau _2 + \mu }} - (d - b)M(t). \end{aligned}$$

It is not difficult to check that there is $t_2> t_1,$ such that

$$\begin{aligned} \mathop M(t) \le \frac{{\lambda _1 \left( {\frac{{(1 - \theta )\varpi \mathop E\limits ^\_ }}{{\tau _1 + \mu + d_1 }} + \varepsilon ^*} \right) + \lambda _2 \left( {\frac{{\theta \rho \mathop E\limits ^\_ }}{{\tau _2 + \mu }} + \varepsilon *} \right) }}{{d - b}} + \varepsilon ^*. \end{aligned}$$

Hence, when $t \ne nT (n \in N),$ and $t > t_2,$ we can obtain

$$\begin{aligned}{} & {} \dot{S}(t)\ge \Pi - g_1 S(t),\\{} & {} \quad g_1 = \mu + \frac{{\beta \mathop E\limits ^\_ }}{K} + \eta \mathop E\limits ^\_ \left( \varepsilon ^*+ {\frac{{\lambda _1 \left( {\frac{{(1 - \theta )\varpi \mathop E\limits ^\_ }}{{\tau _1 + \mu + d_1 }} + \varepsilon ^*} \right) + \lambda _2 \left( {\frac{{\theta \rho \mathop E\limits ^\_ }}{{\tau _2 + \mu }} + \varepsilon ^*} \right) }}{{d - b}} } \right) . \end{aligned}$$

For the case of $n_{10} T > t_2$ and $t> n_{10} T,$

25 $$\begin{aligned} \left\{ {\begin{array}{*{20}c} {\dot{v}(t) \ge \Pi - g_1 v(t),t \ne nT} \\ {v(t^ + ) = v(t)(1 - \theta _1 ),t = nT,} \\ \end{array}} \right. \begin{array}{*{20}c} &{} {n > n_{10} } \\ \end{array}, n \in N^ +. \end{aligned}$$

It follows from Lemma 1 that

$$\begin{aligned} {\overline{v}} (t) = - e^{ - g_1 (t - nT)} \mathrm{{(}}\frac{\Pi }{{g_1 }} - v^* ) + \frac{\Pi }{{g_1 }},t \in (nT,(n + 1)T] \end{aligned}$$

is globally attractive, in which

$$\begin{aligned} v^* = \frac{{\Pi (1 - e^{ - g_1 T} )(1 - \theta _1 )}}{{(1 - (1 - \theta _1 )e^{ - g_1 T} )g_1 }}. \end{aligned}$$

Thus, there exists a constant $t_3 > t_2,$ satisfying that when $t> t_3,$

26 $$\begin{aligned} S(t) \ge \mathop v\limits ^\_ (t) - \varepsilon ^* \ge v^* - \varepsilon ^*\mathop = \limits ^\Delta S^L. \end{aligned}$$

According to (26) and (3), we can obtain

27 $$\begin{aligned} \dot{E}(t) \ge {\left( {\beta \frac{{S^L }}{{S^L + K}} - ((1 - \theta )\varpi ) + \theta \rho + \mu } \right) E(t)}. \end{aligned}$$

Integrating (27) from *nT* to $(n+1)T,$ considering that $\varepsilon ^*$ is arbitrary small and ${\bar{E}}>0$, then we can get

$$\begin{aligned} \begin{array}{l} \mathop E\limits ((n + 1)T) \ge \begin{array}{*{20}c} {E(nT)e^{\left( {\beta \frac{{S^L }}{{S^L + K}} - ((1 - \theta )\varpi + \theta \rho + \mu )} \right) } } &{} {} \\ \end{array} \\ \begin{array}{*{20}c} {\begin{array}{*{20}c} {} &{} {} &{} {\begin{array}{*{20}c} {} &{} {} \\ \end{array}} &{} \ge \\ \end{array}} &{} {E(nT)e^{^{\left( {\beta \frac{\zeta }{{\zeta + K}} - ((1 - \theta )\varpi + \theta \rho + \mu )} \right) } } } &{} {}. \\ \end{array} \\ \end{array} \end{aligned}$$

Using $R_{1*} > 1,$ we get

$$\begin{aligned} \beta \frac{\zeta }{{\zeta + K}} - ((1 - \theta )\varpi + \theta \rho + \mu )>0. \end{aligned}$$

Thus

$$\begin{aligned} \begin{array}{l} E((n_0 + k)T) \\ \ge \begin{array}{*{20}c} {\left( {e^{\beta \frac{\zeta }{{\zeta + K}} - ((1 - \theta )\varpi + \theta \rho + \mu )} } \right) ^kE(n_0 T) \rightarrow \infty ,k \rightarrow \infty ,n_0 T > t_3. } \\ \end{array} \\ \end{array} \end{aligned}$$

This is the contradiction with that *E*(*t*) is bounded. Thus, there is $t^* > t_0,$ such that $E(t^*) \ge \mathop E\limits ^\_,$ and the above prediction is proved.

### Theorem 2

When $R_{1*} > 1,$ for system (3), there will be $q_2$ satisfying that there exists some positive solution which satisfies $q_2\le E(t).$

### Proof

Define

$$\begin{aligned} q_2 = \mathop E\limits ^\_ e^{ - ((1 - \theta )\varpi + \theta \rho + \mu )T}. \end{aligned}$$

According to Lemma 2, $\left( {S(t),E(t),I(t),A(t),N(t),M(t)} \right)$ is discussed based on the following cases.

Firstly, $E(t) \ge \mathop E\limits ^\_$ when *t* takes sufficiently large value.

Secondly, *E*(*t*) vibrates about $\mathop E\limits ^\_$ when *t* takes sufficiently large value.

For case 1, we have $\mathop {\lim }\limits _{t \rightarrow \infty } \inf E(t)> q_2 .$ Immediately, we have the conclusion of Theorem 2.

For case 2, let $t_4> t_3$ and $t_5$ be sufficiently large satisfying

$$\begin{aligned} E(t_4 ) = E(t_5 ) = {\bar{E}}, E(t) < {\bar{E}},\forall t \in (t_4,t_5 ). \end{aligned}$$

If $$t_5- t_4\le T$$, since

$$\begin{aligned} E(t_4 ) = \mathop E\limits ^\_, \end{aligned}$$

and

$$\begin{aligned} -E(t)(\mu +\theta \rho +(1-\theta )\varpi )\le \dot{E}(t). \end{aligned}$$

we have $$E(t) \ge q_2$$ ($$t\in [t_4,t_5 ]$$).

When $$t_5-t_4> T,$$ then

$$\begin{aligned} E(t) \ge q_2 \end{aligned}$$

if $$t \in [t_4,t_5]$$.

Similarly, we can prove

$$\begin{aligned} E(t)\ge q_2 \end{aligned}$$

when $$t \in [t_4,t_4 + T]$$ and

$$\begin{aligned} S^L<S(t) \end{aligned}$$

when $$t \in [t_4 + T,t_5]$$.

In the following, the conclusion that $E(t) \ge q_2$ holds true when $t_4 + T\le t\le t_5,$ will be proved.

Herein we use the method of contradiction, and it means there is a positive constant $T^\prime$ satisfying $q_2\le E(t)$ if $t_4\le t\le T'+ T+t_4,$

$$\begin{aligned} E(T'+ T+t_4) = q_2 \end{aligned}$$

and

$$\begin{aligned} \dot{E} (t_4 + T + T') \le 0. \end{aligned}$$

Exploiting (3), and $t =T+T'+t_4$, then

$$\begin{aligned} \begin{array}{*{20}l} {\dot{E}(t) = \eta E(t)M(t)S(t) - \varpi (1 - \theta )E(t) + \frac{{\beta S(t)}}{{S(t) + K}} - \theta \rho E(t) - \mu E(t)} \\ { \ge q_2 \left( {\beta \frac{{S^L }}{{S^L + K}} - (1 - \theta )\varpi - \theta \rho - \mu } \right) > 0}. \\ \end{array} \end{aligned}$$

This is a contradiction. So $E(t) \ge q_2$ when $t \in [t_4 ,t_5].$

### Theorem 3

If $R_{1*} > 1,$ we have the conclusion that model (3) is permanent.

### Proof

We assume the arbitrary solution of (3) is denoted by $\left( {S(t),E(t),I(t),A(t),N(t),M(t)} \right) .$

If *t* takes a sufficiently large value, we can obtain

$$\begin{aligned} \dot{S}(t) \ge \Pi - S(t) \left( \mu +\frac{\Pi \beta }{{\mu K}} + \eta \frac{{\Pi ^2 }}{{\mu ^2 }}\right) . \end{aligned}$$

Using the same method, we have

$$\begin{aligned} \mathop {\lim }\limits _{t \rightarrow \infty } S(t) \ge q_1, \end{aligned}$$

where

$$\begin{aligned} q_1 = \frac{\Pi }{{\beta \frac{\Pi }{{\mu K}} + \eta \frac{{\Pi ^2 }}{{\mu ^2 }} + \mu }}\frac{{(1 - \theta _1 )\left( {e^{\left( {\beta \frac{\Pi }{{\mu K}} + \eta \frac{{\Pi ^2 }}{{\mu ^2 }} + \mu } \right) T} - 1} \right) }}{{e^{\left( {\beta \frac{\Pi }{{\mu K}} + \eta \frac{{\Pi ^2 }}{{\mu ^2 }} + \mu } \right) T} + \theta _1 - 1}} - \varepsilon ^* \end{aligned}$$

($\varepsilon ^*$ is sufficiently small).

When *t* takes a sufficiently large value, we derive

$$\begin{aligned} \dot{I}(t) \ge (1 - \theta )\varpi q_1 - (\tau _1 + \mu + d_1 )I(t). \end{aligned}$$

Therefore

$$\begin{aligned} \mathop {\lim }\limits _{t \rightarrow \infty } I(t) \ge q_3, \end{aligned}$$

where

$$\begin{aligned} q_3 = \frac{{(1 - \theta )\varpi q_1 }}{{(\tau _1 + \mu + d_1 )}}. \end{aligned}$$

When *t* is sufficiently large, we can obtain

$$\begin{aligned} \dot{A}(t) \ge \theta \rho q_1 - A(t)(\tau _2 + \mu _1 ). \end{aligned}$$

So

$$\begin{aligned} \mathop {\lim }\limits _{t \rightarrow \infty } A(t) \ge q_4, \end{aligned}$$

where

$$\begin{aligned} q_4 = \frac{{\theta \rho q_1 }}{{\tau _2 + \mu _1}}. \end{aligned}$$

According to Theorem 2, when *t* takes sufficiently large value, then

$$\begin{aligned} \left\{ {\begin{array}{*{20}l} {\dot{M}(t) \ge - (d - b)M(t)+ \lambda _2 q_4+q_3\lambda _1} \\ {M(t^ + ) = M(t)(1 - \xi )}. \\ \end{array}} \right. \end{aligned}$$

Therefore

$$\begin{aligned} \mathop {\lim }\limits _{t \rightarrow \infty } M(t) \ge q_5, \end{aligned}$$

where

$$\begin{aligned} q_5 = \frac{{\lambda _1 q_3 + \lambda _2 q_4 }}{{b - d}}\frac{{\left( {1 - e^{T\left( {d - b} \right) } } \right) (1 - \xi )}}{{e^{\left( {d - b} \right) T} + \xi - 1}} - \varepsilon ^* \end{aligned}$$

( $\varepsilon ^*$ is sufficiently small).

Set

$$\begin{aligned} \begin{array}{l} \Omega _0 = \bigg \{ \left( {S(t),E(t),I(t),A(t),N(t),M(t)} \right) \in R_ + ^6:I(t) \ge q_3,A(t) \ge q_4,N(t) \ge 0, \\ \;\;\;\;\;\;\;\;\;\;\;\;E(t) \ge q_2,S(t) \ge q_1,M(t) \le \frac{{\Pi (\lambda _1 + \lambda _2 )}}{{\mu (d - b)}},d > b,\\ \frac{\Pi }{\mu }\ge N(t)\ge A(t)+I(t)+E(t)+S(t), M(t) \ge q_5 \bigg \}. \end{array} \end{aligned}$$

It follows from Theorem 3 and the analysis above that $\Omega _0$ belongs to the globally attractive region of $\Omega .$ For the system (3), the positive solution satisfying the condition (*) will finally enter and remain in $\Omega _0,$ i.e., the system will be permanent.

According to Theorem 2 and Theorem 3, the following assertions can be easily obtained.

### Proposition 3

If $\theta _1 < \theta _1^*$, system (3) will be permanent, i.e., the Gompertz virus will be an endemic disease, in which

$$\begin{aligned} \begin{array}{*{20}c} {\theta _1^* = \frac{{\left\{ {\Pi \beta - \left( {\Pi + K\mu } \right) [(1 - \theta )\varpi + \theta \rho + \mu ]} \right\} (e^{\mu T} - 1)}}{{K\mu [(1 - \theta )\varpi + \theta \rho + \mu ] + \Pi [\beta - (1 - \theta )\varpi + \theta \rho + \mu ](e^{\mu T} - 1)}}} \\ {\left( {\Pi \beta > \left( {\Pi + K\mu } \right) [(1 - \theta )\varpi + \theta \rho + \mu ]} \right) } \end{array}. \end{aligned}$$

When $T > \mathop T\limits ^\_$, system (3) will be permanent, and the Gompertz virus will be an endemic disease, in which

$$\begin{aligned}\bar{ T }= \frac{1}{\mu }\ln \frac{{(1 - \theta _1 )[k\alpha \mu - (\beta - \alpha )\Pi ]}}{{k\alpha \mu - \Pi (\beta - \alpha )(1 - \theta _1 )}},\alpha = (1 - \theta )\varpi + \theta \rho + \mu . \end{aligned}$$

It follows from Theorem 1 and Theorem 3 that the Gompertz virus will not appear when $R_1^*< 1$, and when $R_{1*}>1,$ the Gompertz virus will be uniformly persistent.

## Numerical simulations

In this section, the Runge-Kutta method is exploited to perform numerical simulations of Gompertz virus disease model with impulsive effect. Numerical simulations are presented to validate the analyzed conclusion from the view of impulsive differential equation theory.

**Figure 1:**
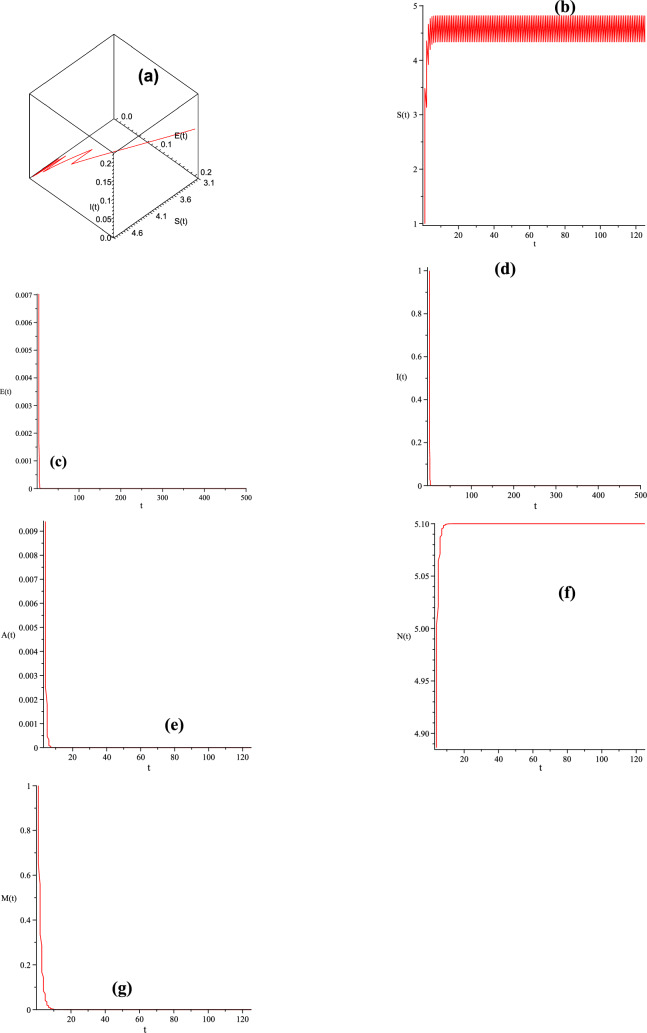
Dynamical behavior of system (3) with $\beta =0.1, \eta =0.2, \lambda _1=0.1, \Pi = 5.1, \mu =1, b=0.3, \lambda _2=0.15, \rho = 1, T= 1, d= 1, K= 2, \varpi = 1, \tau _1= 1.5, \tau _2= 1, \xi = 0.02, \theta _1=0.1, \theta =0.5, d_1= 0.2,$ and all the parameters satisfy $R_1^*=0.6498<1.$ (**a**) (*I*(*t*), *E*(*t*), *S*(*t*));  (**b**) trajectory of the susceptible pest *S*(*t*);  (**c**) trajectory of the exposed pest *E*(*t*);  (**d**) trajectory of the infected pest *I*(*t*);  (**e**) trajectory of the asymptotically infected pest *A*(*t*);  (**f**) trajectory of the total population of pest *N*(*t*);  (**g**) trajectory of the reservoir or the seafood place or market *M*(*t*).

**Figure 2:**
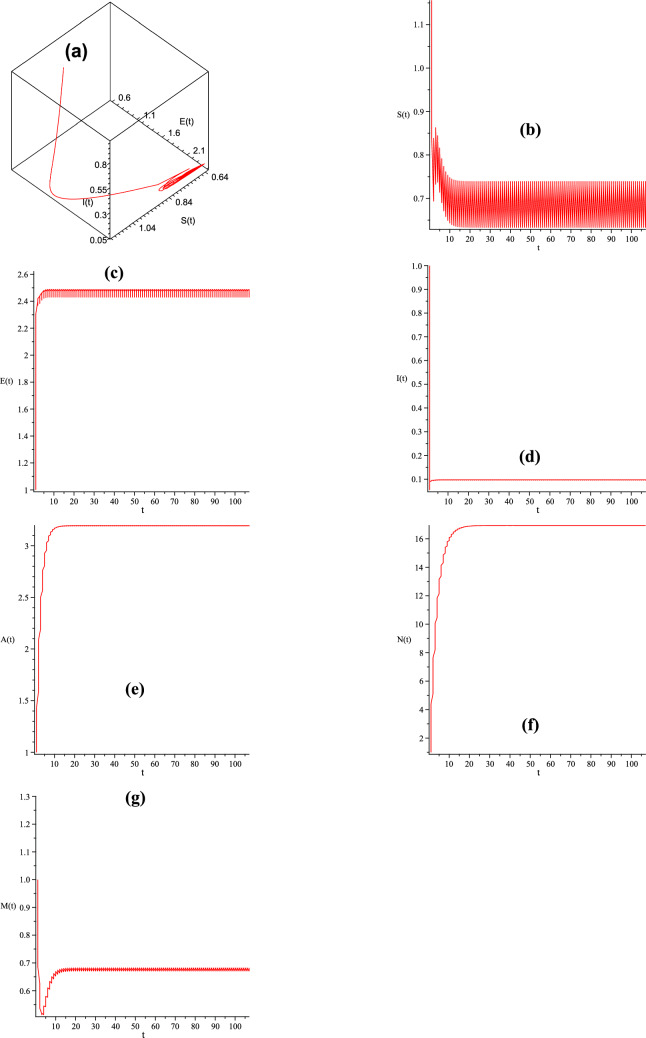
Dynamical behavior of system (3) with $\beta =5, \eta =2, \lambda _1=0.1, \Pi = 5.1, \mu =0.3, b=0.3, \lambda _2=0.15, \rho =1.3, T= 1, d= 1, K=3, \varpi = 2, \tau _1= 25, \tau _2=0.2, \xi = 0.02, \theta _1=0.13, \theta =0.5, d_1= 0.2,$ and all the parameters satisfy $R_{1*}=2.0644>1.$ (**a**) (*I*(*t*), *E*(*t*), *S*(*t*));  (**b**) trajectory of the susceptible pest *S*(*t*);  (**c**) trajectory of the exposed pest *E*(*t*);  (**d**) trajectory of the infected pest *I*(*t*);  (**e**) trajectory of the asymptotically infected pest *A*(*t*);  (**f**) trajectory of the total population of pest *N*(*t*);  (**g**) trajectory of the reservoir or the seafood place or market *M*(*t*).

Firstly, when we choose $\Pi = 5.1, \lambda _1=0.1, \eta =0.2, \beta =0.1, \mu =1, b=0.3, \lambda _2=0.15, \rho = 1, T= 1, d= 1, K= 2, \varpi = 1, \tau _1= 1.5, \tau _2= 1, \xi = 0.02, \theta _1=0.1, \theta =0.5, d_1= 0.2,$ we compute $R_1^*=0.6498<1,$ which satisfies the condition of Theorem 1. By Theorem 1, the infection-free periodic solution of (3) is globally asymptotically stable. Figure 1 also demonstrates this conclusion: The periodic solution has the characteristic of global attractivity. It can be shown from Fig. 1 that *S*(*t*) goes to oscillatory; *E*(*t*),  *I*(*t*),  *A*(*t*) and *M*(*t*) go to extinction; *N*(*t*) goes to some stable value. It implies that the susceptible pest population oscillate with a positive amplitude, i.e., they will not go extinct. Additionally, the infective pests almost will go extinct and have little effect on the crop, which can be shown in Fig. 1.

Moreover, when we choose $\Pi = 5.1, \lambda _1=0.1, \eta =2, \beta =5, \mu =0.3, b=0.3, \lambda _2=0.15, \rho =1.3, T= 1, d= 1, K=3, \varpi = 2, \tau _1= 25, \tau _2=0.2, \xi = 0.02, \theta _1=0.13, \theta =0.5, d_1= 0.2,$ we compute $R_{1*}=2.01>1,$ which satisfies the condition of Theorem 3. By Theorem 3, we can obtain that the proposed model is permanent, which can be shown in Fig. 2. Figure 2 also demonstrates this conclusion: The periodic solution has the characteristic of permanence. It can be shown in Fig. 2 that *M*(*t*),  *A*(*t*),  *I*(*t*),  *E*(*t*) and *S*(*t*) go to oscillatory; *N*(*t*) goes to some stable value. It means that all the species will not go extinct and will almost maintain a stable value, which can be shown in Fig. 2.

From the view of ecology, infective pests almost have no effect on crops, but the pests that have a significant impact on the crops will become extinct. This will also demonstrate the effectiveness of scientific control. Actually, the purpose of scientific control is not to eradicate pests, but to control the number of pests to a certain level.

## Conclusions

A new mathematical biological model on the Gompertz virus impulsive system is proposed in this paper. This proposed model considers the factors of the recovered or the removed population, asymptotically infected, infected (symptomatic), exposed and susceptible population. Utilizing the Floquet theory, we strictly prove the infection-free periodic solution of the proposed model is globally attractive if $R_1^* < 1.$ It means that the infective pests almost will go extinct and have little effect on the crop. Moreover, we also obtain that the system (3) is permanent if $R_{1*}> 1.$ Hence, we exploit the impulsive control method based on the effect of the Gompertz virus on pest such that $R_1^* < 1,$ and then drive the virus to extinction. In addition, we can also control that the susceptible, exposed, infected (symptomatic), asymptotically infected population and the Gompertz virus in the market or seafood place or the reservoir oscillate with a positive amplitude. Actually, the scientific impulsive control is not to eradicate pests, but to control the number of pests to a certain level. Therefore, the proposed impulsive control strategy in this paper is very effective in impulsive control for the Gompertz virus model. Considering that time delays unavoidably exist in the transmission of the impulsive information and sampling in lots of practical cases, the proposed system will be extended to the delayed impulsive control for the Gompertz virus disease model in the future.

## Data Availability

Data will be made available on request from the corresponding author (Youxiang Xie).
